# Early presentations of dementia in a diverse population

**DOI:** 10.1002/alz.14578

**Published:** 2025-02-26

**Authors:** Sedigheh Zabihi, Jonathan P Bestwick, Mark Jitlal, Phazha LK Bothongo, Qiqi Zhang, Christine Carter, Moïse Roche, Sarah Morgan‐Trimmer, Yvonne Birks, Mark Wilberforce, Ruth Dobson, Alastair J Noyce, John Robson, Fiona M Walter, Claudia Cooper, Charles R Marshall

**Affiliations:** ^1^ Centre for Preventive Neurology Wolfson Institute of Population Health Queen Mary University of London London UK; ^2^ Centre for Psychiatry and Mental Health Wolfson Institute of Population Health Queen Mary University of London London UK; ^3^ Division of Psychiatry University College London London UK; ^4^ Department of Health and Community Sciences Faculty of Health and Life Sciences University of Exeter Exeter UK; ^5^ NIHR School for Social Care Research University of York York UK; ^6^ The London School of Medicine and Dentistry Queen Mary University of London London UK; ^7^ Centre for Cancer Screening Prevention and Early Diagnosis Wolfson Institute of Population Health Queen Mary University of London London UK

**Keywords:** dementia, early diagnosis, primary care, prodromal

## Abstract

**INTRODUCTION:**

Improved recognition of non‐cognitive presentations of dementia could reduce inequalities in dementia diagnosis, particularly if sociocultural factors influence help‐seeking for cognitive symptoms.

**METHODS:**

We conducted a nested case‐control study using electronic healthcare records from primary care practices in East London, United Kingdom, to assess associations between prediagnostic presentations to primary care and subsequent dementia diagnosis.

**RESULTS:**

We included 4137 individuals with a dementia diagnosis and 15,754 controls in the matched analysis. In addition to memory difficulties, a range of symptoms were more common in the decade before diagnosis, including depression, anxiety, use of antipsychotics, insomnia, constipation, incontinence, hypotension, hearing loss, imbalance, and dizziness.

**DISCUSSION:**

A range of non‐cognitive presentations are seen during the prodromal period of dementia in a diverse population. Improved recognition of these associations and their variation by ethnicity could increase access to dementia diagnosis through improved recognition of early features in people from different sociocultural backgrounds.

**Highlights:**

- Prediagnostic signs of dementia include cognitive and non‐cognitive symptoms.
- Psychiatric symptoms are common up to a decade prior to a dementia diagnosis.
- Autonomic prediagnostic symptoms are more common among South Asian groups.
- The importance of prediagnostic symptoms of dementia varies by ethnicity.

## 1 BACKGROUND

Timely diagnosis of dementia empowers people living with dementia to understand their symptoms, plan for the future, and access current, symptomatic treatments. Timely diagnosis could also improve access to emerging disease‐modifying treatments.^1^

People from certain ethnic backgrounds may report physical or affective difficulties more readily than cognitive changes.^2^, ^3^, ^4^ This could be due to the stigma associated with memory difficulties and receiving a dementia diagnosis in certain cultures, distrust in healthcare systems, and sociocultural factors impacting the way people experience their symptoms or seek help for them.^5^ Recognition of the non‐cognitive signs and symptoms people experience in the years before receiving a dementia diagnosis could potentially help mitigate diagnostic inequities in dementia.

Early features and prediagnostic presentations of dementia have been identified through large, population‐based observational studies.^6^, ^7^, ^8^, ^9^ However, these populations have been predominantly White and relatively affluent, and previous research on Parkinson's disease (PD) revealed substantial differences in early presentations to primary care when a more diverse and deprived population was studied.^10^ We aimed to investigate patterns of presentation to primary care prior to dementia diagnosis in a diverse and deprived inner‐city population with universal access to healthcare. We hypothesized that the recording of various neuropsychiatric, autonomic, and sensory symptoms would be more common among those who subsequently received a dementia diagnosis and that, due to sociocultural factors, they would be more common among ethnic minority groups.

## 2 METHODS

### 2.1 Design and setting

We performed a nested case‐control study in a large primary care data set in East London, United Kingdom (UK). In the UK National Health Service (NHS), primary care records represent accumulated medical information about patients throughout their lifetime. If patients move to the UK, a medical history is taken and entered into the notes and cross‐checked with medical notes if available. The primary care database was derived from electronic health records (1990 to 2018) in East London, which included 1,016,277 individuals (98% of all adults in the region) from general practices across four clinical commissioning groups in East London: Hackney & City of London, Newham, Tower Hamlets, and Waltham Forest. The NHS Health Research Authority waived the need for ethical approval due to the anonymized nature of the dataset. We followed the Strengthening the Reporting of Observational Studies in Epidemiology (STROBE) reporting guideline^11^ in this study.

### 2.2 Participants

#### 2.2.1 Identification of cases and controls

All individuals with a coded and dated diagnosis of dementia were included as cases (S1). Patients with dementia missing a diagnosis date were excluded. Controls were selected from those without a code of dementia or other chronic neurological condition, including PD, multiple sclerosis, and motor neuron disease, and were matched to cases on age and sex with the ratio 4:1. Controls were assigned an index date, which was the date of dementia diagnosis of the case to which they were matched. Index date or dementia diagnosis date was used to categorize the time periods for prediagnostic exposures.

**RESEARCH IN CONTEXT**

1. **Systematic review**: The authors systematically reviewed the evidence on non‐cognitive symptoms people experience prior to receiving a dementia diagnosis and assessed the quality of existing evidence. The majority of previous studies were conducted in affluent areas, which contained less diverse populations.
2. **Interpretation**: This study describes the results of a nested case‐control study comparing prediagnostic symptoms in people with dementia and their matched controls in a diverse population in East London. Among 4137 individuals with dementia and 15,754 matched controls, the study found that memory difficulties and neuropsychiatric, autonomic, and sensory symptoms were recorded more frequently up to 10 years before diagnosis. There was evidence of ethnocultural variation in the magnitude of association for a majority of these symptoms.
3. **Future directions**: Improved recognition of non‐cognitive presentations to primary care in years before receiving a dementia diagnosis and their association with ethnicity can lead to earlier and more equitable dementia diagnosis for people from diverse backgrounds.

#### 2.2.2 Exposure selection and data extraction

Prediagnostic presentations of dementia were defined as signs and symptoms encountered in primary care up to 10 years prior to receiving a dementia diagnosis. They were selected based on a review and meta‐analysis of the literature,^6^ qualitative interviews with people living with dementia from minoritized ethnicities in East London,^5^ and consulting with public and patient collaborators and clinical and academic team members. Nineteen exposures (Table S1) were selected and categorized into (1) neuropsychiatric symptoms (eg, depression, anxiety), (2) autonomic symptoms (eg, fatigue, constipation), (3) sensory (eg, hearing loss), and (4) cognitive symptoms (memory difficulties). Individual patient information was extracted by the Clinical Effectiveness Group at Queen Mary University of London on February 6, 2018. All exposures were recorded up to twice in the database (earliest ever record and most recent record for that exposure). If there was more than one observation, the earliest date was used for the analysis.

Given the cross‐sectional nature of data extraction, calculation of true incidence rates was not possible. Therefore, previously developed methods were used to examine temporal relationships^10^ using three intervals to evaluate exposure–outcome associations (<2, 2 to <5, and 5 to 10 years before dementia diagnosis or index date). Exposure variables with <1% prevalence among individuals with dementia across all time periods were excluded.

#### 2.2.3 Exposure definition

The variables extracted were based on identified data comprising diagnoses, laboratory test results, and demographic details coded using the Read coding system (https://digital.nhs.uk/services/terminologyand‐classifications/read‐codes) (Table S2and S3). Variables were defined in a way that maximized information use for the modeling. For this reason, missing data were categorized as unknown rather than being excluded. Rates of missingness were low (<1% for all exposures).

The age of the participants was calculated based on the date of data extraction (February 6, 2018). Ethnicity was defined by the self‐reported UK Census categories, grouped here into five groups: Black (African, Caribbean, and other Black), South Asian (Bangladeshi, Indian, and Pakistani), White (British, Irish, and other White), Other (Chinese, other, and mixed groups), and unknown. The Index of Multiple Deprivation (IMD) is a global area‐based measure of socioeconomic status, based on seven categories including income, employment, educational level, health, crime, housing, and environment. Raw IMD scores were assigned to deciles derived from national data and converted into quintiles. Quintile 1 (IMD 1‐2) represented the 20% of the population with the most socioeconomic deprivation, and quintile 5 (IMD 9‐10) represented the 20% of the population with the least socioeconomic deprivation. The IMD group 1‐2 was used as the reference category in the analyses.

All symptoms were defined on four levels: whether the symptom was never recorded, first recorded before the dementia diagnosis (or the index date for controls), first recorded after dementia diagnosis, or unknown if date was missing. For each symptom, the status was determined by the early record, unless this record was missing, in which case the status at the late record was used. For most symptoms, including depression, anxiety, insomnia, constipation, incontinence, hypotension, fatigue, erectile dysfunction, imbalance, memory difficulties, and dizziness, we used the coded diagnosis. Due to the unavailability of a specified coded diagnosis of psychosis or psychotic symptoms, we used the recorded use of antipsychotics as a proxy. The date of first prescription was used to categorize the exposure. Hearing loss was defined by a coded diagnosis or a referral for assessment due to reported hearing difficulty. Similarly, coded diagnoses of neck and shoulder pain were combined to define musculoskeletal pain.

### 2.3 Statistical analysis

Data analysis was performed between June 2023 and March 2024. For the periods from less than 2 years, 2 to 5 years, and 5 to 10 years before the index date (date of diagnosis), we calculated the overall occurrence of prediagnostic symptoms as the absolute number and percentage. A matched case‐control cohort was selected by matching four controls for each individual with dementia according to age (calendar year) and sex. We then ran conditional logistic regressions including cases and controls, adjusted for age and sex. We used this categorization to estimate the odds ratio (OR) for subsequent dementia for each variable of interest in each period and in all three periods combined. We then ran a sensitivity analysis adjusting for deprivation (IMD) level. Furthermore, we included the entire population aged over 65 without a chronic neurological disease in an unmatched multivariable logistic regression. The estimate for each exposure of interest was stratified by ethnicity and adjusted for age and sex. We then ran an interaction test (with the White group as the reference) for the entire 10‐year period prior to diagnosis to investigate whether there was a significant difference in estimates for each exposure across different ethnicities. Where there was evidence of an interaction with ethnicity, we calculated the proportion of cases presenting each symptom during the 10‐year period before the dementia diagnosis in each ethnicity category and performed chi‐squared tests.

For exposures that we found to be associated with subsequent dementia diagnosis, we calculated the positive predictive values (PPVs) for dementia up to 10 years following the exposure using the dementia patients and the entire population aged over 65 without exclusion criteria (a chronic neurological disease other than dementia).

The analyses were performed using R version 4.1.2,^12^ Stata versions 17 and 18,^13^ and MATLAB.^14^

## 3 RESULTS

### 3.1 Demographic characteristics

From 1,016,277 individuals included in the dataset, we included 4137 individuals with a dementia diagnosis and 15,754 matched controls for the case‐control analysis. Demographic characteristics of the sample are summarized in Table 1. People with a dementia diagnosis and controls were comparable in age (mean age for cases [SD] = 81.3 [8.8]; controls: 80.7 [SD] = 8.6) and sex (female cases = 60.3%; female controls = 59.4%). The ethnic composition of the population was consistent with UK Census estimates for the region (Black = 13.4%; South Asian = 21.4%; White = 43.8%; other ethnicities = 11.3%), with 10.1% of the population missing data on their ethnicity. There were high levels of deprivation, with most participants (85%) living in the four most deprived deciles of the UK (IMD 1‐4).

**TABLE 1:** Demographic characteristics of the sample.

	Cases	Matched controls	Total	
Characteristic	*N* = 4137	*N* = 15,754	*N* = 1,016,277	*P*
Gender, n(%)				.29
Female	2494 (60.3%)	9354 (59.4%)	496,577 (48.7%)	
Male	1643 (39.7%)	6400 (40.6%)	519,686 (51.13%)	
Unknown	0	0	14 (< 0.1)	
Age, mean (SD)	81.31 (8.85)	80.73 (8.65)	40.48 (15.42)	.85
Ethnicity, *n* (%)				<.001
White	1965 (47.5%)	8336 (52.91%)	444,931 (43.78%)	
Black	941 (22.75%)	2828 (17.95%)	135,971 (13.38%)	
South Asian	685 (16.56%)	2547 (16.17%)	217,803 (21.43%)	
Other	281 (6.79%)	1198 (7.60%)	114,995 (11.32%)	
Unknown	265 (6.41%)	845 (5.36%)	102,577 (10.09%)	
IMD, *n* (%)				<.001
1–2 (most deprived)	2115 (51.12%)	7091 (45.01%)	457,061 (44.97%)	
3–4	1347 (32.56%)	6352 (40.32%)	427,322 (42.05%)	
5–6	190 (4.59%)	1240 (7.87%)	77,163 (7.59%)	
7–8	58 (1.40%)	274 (1.74%)	19,376 (1.91%)	
9–10 (least deprived)	27 (0.65%)	153 (0.97%)	6964 (0.69%)	
Unknown	400 (9.67%)	644 (4.09%)	28,391 (2.79%)	

*Note*: Demographic characteristics of the cases and controls in addition to all the population shown in numbers (*n*) and percentages (%) and mean and standard deviation.

Abbreviations: IMD, Index of Multiple Deprivation; SD, standard deviation.

### 3.2 Prediagnostic manifestations

Table 2 summarizes the prediagnostic manifestations in the three periods from the matched case‐control analysis. Patients who were prescribed antipsychotics had increased odds of receiving a dementia diagnosis within 2 years (OR = 12.7, 95% CI: 9.01 to 17.90), 2 to 5 years (OR = 3.88, 95% CI: 2.64 to 5.70), and 5 to 10 years (OR = 3.13, 95% CI: 2.22 to 4.42) prior to the diagnosis. Similarly, depression and anxiety were associated with subsequent dementia diagnosis across all time periods. However, insomnia was only found to be associated with receiving a dementia diagnosis within 2 years (OR = 1.69, 95% CI: 1.31 to 2.17).

**TABLE 2:** Matched case‐control analysis of prediagnostic signs and symptoms.

	Time period							
	<2		2 to <5 years		5 to 10 years		<10 years	
Category	*N* (%) (Dementia: control)	OR (95% CI)	*N* (%) (Dementia: control)	OR (95% CI)	*N* (%) (Dementia: control)	OR (95% CI)	*N* (%) (Dementia: control)	OR (95% CI)
**Prediagnostic signs and symptoms**								
***Neuropsychiatric***								
Depression	112 (2.71):76 (0.48)	6.18 (4.60 to 8.31)***	73 (1.76):124 (0.79)	2.53 (1.88 to 3.41)***	112 (2.71):265 (1.68)	1.79 (1.43 to 2.25)***	297 (7.18):465 (2.95)	2.71 (2.33 to 3.16)***
Anxiety	115 (2.78): 115 (0.98)	3.03 (2.37 to 3.87)***	73 (1.76): 217 (1.38)	1.34 (1.02 to 1.76)*	123 (2.97): 379 (2.41)	1.31 (1.07 to 1.62)**	311 (7.52):751 (4.77)	1.67 (1.46 to 1.92)***
Use of antipsychotics	133 (3.21): 49 (0.31)	12.7 (9.01 to 17.90)***	51 (1.23): 60 (0.38)	3.88 (2.64 to 5.70)***	58 (1.40): 87 (0.55)	3.13 (2.22 to 4.42)***	242 (5.85):196 (1.24)	5.84 (4.80 to 7.11)***
Insomnia	89 (2.15): 210 (1.33)	1.69 (1.31 to 2.17) ***	91 (2.20): 324 (2.06)	1.07 (0.85 to 1.36)	191 (4.62): 630 (4)	1.16 (0.98 to 1.38)	371 (8.97):1164 (7.39)	1.23 (1.09 to 1.40)**
***Autonomic***								
Fatigue	70 (1.69): 298 (1.89)	0.90 (0.69 to 1.17)	107 (2.59): 356 (2.26)	1.14 (0.91 to 1.42)	161 (3.89): 498 (3.16)	1.26 (1.05 to 1.42)	338 (8.17):1152 (7.31)	1.13 (0.99 to 1.28)
Constipation	162 (3.92): 411 (2.61)	1.68 (1.40 to 2.02)***	191 (4.62): 592 (3.76)	1.38 (1.16 to 1.63)***	266 (6.43): 722 (4.58)	1.57 (1.36 to 1.83)***	619 (14.96):1725 (10.95)	1.54 (1.39 to 1.71)***
Erectile dysfunction	41 (2.50): 171 (2.67)	0.95 (0.67 to 1.34)	88(5.36):289 (4.52)	1.24 (0.96 to 1.59)	122 (7.43): 401 (6.27)	1.24 (1.00 to 1.54)*	251 (15.28):861 (13.45)	1.18 (1.01 to 1.38)*
Incontinence	190 (4.59): 290 (1.84)	3.16 (2.61 to 3.84)***	145 (3.5): 258 (1.64)	2.66 (2.14 to 3.29)***	127 (3.07): 295 (1.87)	2.02 (1.63 to 2.50)***	462 (11.17):843 (5.35)	2.63 (2.32 to 2.97)***
Hypotension	75 (1.81): 89 (0.56)	3.28 (2.40 to 4.49)***	54 (1.31): 111 (0.70)	1.89 (1.35 to 2.63)***	37 (0.89): 110 (0.70)	1.31 (0.90 to 1.91)	166 (4.01):310 (1.97)	2.10 (1.72 to 2.55)***
***Sensory***								
Balance difficulties	153 (3.70): 461 (2.93)	1.30 (1.08 to 1.58)**	216 (5.22): 587 (3.73)	1.43 (1.22 to 1.69)***	197 (4.76): 640 (4.06)	1.22 (1.03 to 1.44)*	566 (13.68):1688 (10.71)	1.31 (1.18 to 1.46)***
Dizziness	180 (4.35): 489 (3.10)	1.46 (1.22 to 1.74)***	229 (5.54): 689 (4.37)	1.30 (1.11 to 1.52)**	287 (6.94): 987 (6.27)	1.15 (1.00 to 1.32)*	696 (16.82):2165 (13.74)	1.26 (1.15 to 1.39)***
Musculoskeletal pain	96 (2.32):406 (2.58)	0.88 (0.70 to 1.10)	158 (3.82):711 (4.51)	0.81 (0.68 to 0.97)*	301 (7.28):1222 (7.78)	0.91 (0.80 to 1.04)	555 (13.42):2339 (14.85)	0.87 (0.79 to 0.97)*
Hearing loss	178 (4.30):494 (3.14)	1.36 (1.14 to 1.63)**	154 (3.72):530 (3.36)	1.07 (0.89 to 1.29)	164 (3.96):584 (3.71)	1.05 (0.88 to 1.26)	496 (11.99):1608 (10.21)	1.15 (1.03 to 1.29)*
***Cognitive***								
Memory difficulties	1771 (42.81): 310 (1.97)	89.63 (73.82 to 108.83)***	365 (8.82): 204 (1.29)	24.80 (19.69 to 31.24)***	125 (3.02): 108 (0.69)	13.85 (10.04 to 19.10)***	2261 (54.65):622 (3.95)	51.00 (43.87 to 59.29)***

*Note*: The first column shows the proportion of people with dementia experiencing the prediagnostic symptom to the proportion of controls experiencing the same symptom. The association between experiencing signs and symptoms and receiving a dementia diagnosis within 2, 2 to 5, and 5 to 10 years later is shown in ORs (95% CIs). Total *N* for erectile dysfunction: 8043; *p* values represent the statistical significance of the associations: *p < .05; **p < .01; ***p < .001.

Abbreviations: CI, confidence interval; OR, odds ratio.

Hypotension was found to be more frequent among people who went on to be diagnosed with dementia compared to controls. In the matched analysis, individuals with hypotension were approximately three times more likely to be diagnosed with dementia compared to controls within 2 years (OR = 3.28, 95% CI: 2.40 to 4.49). This association was also found within 2 to 5 years prior to receiving a dementia diagnosis (OR = 1.89, 95% CI: 1.35 to 2.63). Constipation and incontinence were associated with increased odds of receiving a diagnosis of dementia across all time periods. On the other hand, we found erectile dysfunction to be associated with subsequent dementia diagnosis within 5 to 10 years before the diagnosis (OR = 1.24, 95% CI: 1.00 to 1.54); however, we found no evidence for an association between fatigue and a subsequent dementia diagnosis.

We found hearing loss to be associated with increased odds of receiving a dementia diagnosis within 2 years of diagnosis (OR = 1.36, 95% CI: 1.14 to 1.63). On the other hand, musculoskeletal pain was significantly lower in people who received subsequent dementia diagnosis compared to controls within 2 to 5 years prior to diagnosis (OR = 0.81, 95% CI: 0.68 to 0.97).

Memory difficulty was the most frequently reported manifestation. Within 2 years of dementia diagnosis, people with memory problems had approximately 90‐fold increased odds of a dementia diagnosis (OR = 89.63, 95% CI: 73.82 to 108.83). This association was also found 2 to <5 years before diagnosis (OR = 24.80, 95% CI:19.69 to 31.24) and 5 to 10 years before diagnosis (OR = 13.85, 95% CI: 10.04 to 19.10). Similarly, dizziness and imbalance were associated with increased odds of receiving a dementia diagnosis across all three time periods.

### 3.3 Temporal trends of prodromal features

**FIGURE 1:**
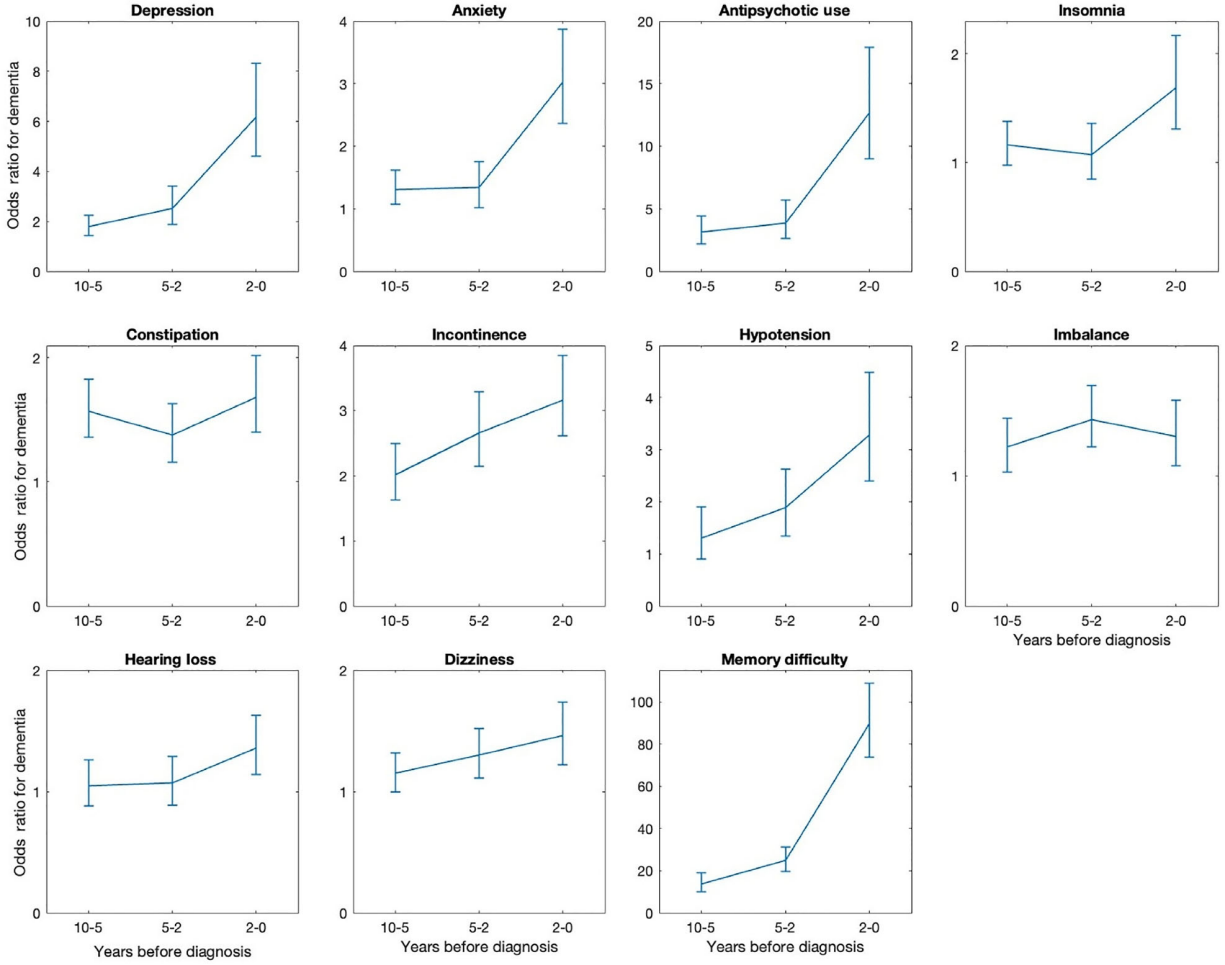
Temporal trends in prediagnostic presentations of dementia. (Error bars are representative of the 95% confidence intervals). Legend: plots show odds of receiving dementia diagnosis within 0 to 2, 2 to 5, and 5 to 10 years after experiencing each prediagnostic symptom.

The temporal trend in the magnitude of association for prediagnostic symptoms significantly associated with subsequent dementia diagnosis is shown in Figure 1. For many features, including memory difficulty, depression, anxiety, antipsychotic use (a marker for presence of psychosis), insomnia, incontinence, hypotension, hearing loss, and dizziness, there was a clear trend whereby the magnitude of the association increased in greater proximity to dementia diagnosis.

### 3.4 Sensitivity analyses

Adjustment for IMD did not materially change the results of the association between each exposure and odds of subsequent dementia diagnosis (Table S4), and results remained consistent after an unmatched sensitivity analysis adjusted for age and sex (Table S5).

### 3.5 Variation by ethnicity

Results of the analysis stratified for ethnicity, including cases and all controls, are summarized in Table S6. We found evidence of an interaction with ethnicity for insomnia, constipation, incontinence, imbalance, hypotension, and memory difficulties. The proportion of people with dementia presenting these symptoms in the 10‐year period prior to dementia diagnosis is summarized in Table 3, stratified by ethnicity. Some symptoms were considerably more prevalent among the South Asian group compared to White and Black groups, including insomnia, constipation, incontinence, imbalance, and memory difficulties. While the proportion of some symptoms was quite similar between White and Black groups, insomnia was more frequently reported in the White groups. Additionally, Black patients reported constipation, incontinence, imbalance, and memory difficulties more frequently than the White group.

**TABLE 3:** Proportion of cases presenting each symptom prior to diagnosis stratified by ethnicity (%).

Symptom	White *N* = 1966	Black *N* = 941	South Asian *N* = 685	Other *N* = 281	Unknown *N* = 265	*P*
Depression	7.93	5.10	7.45	9.25	6.04	<.001
Anxiety	8.85	5.10	7.30	8.54	5.66	<.001
Use of antipsychotics	5.04	6.38	7.74	6.05	4.91	.002
Insomnia	8.49	7.74	13.43	9.96	5.28	<.001
Fatigue	8.90	6.80	9.49	10.68	1.51	<.001
Constipation	13.02	16.68	20.58	15.66	7.92	<.001
Erectile dysfunction	10.78	19.32	24.41	9.09	7.45	<.001
Incontinence	9.72	12.11	16.50	9.96	6.04	<.001
Hypotension	4.53	3.61	3.80	3.91	2.26	.060
Imbalance	12.82	13.28	19.56	14.95	4.91	<.001
Dizziness	15.36	16.15	25.40	18.86	5.66	<.001
Musculoskeletal pain	10.63	12.86	15.33	12.46	6.04	<.001
Hearing loss	13.33	9.67	12.85	12.46	7.55	.008
Memory difficulties	54.93	55.79	62.48	52.31	30.57	<.001

*Note*: Proportion (%) of people with dementia experiencing each symptom in each ethnicity group within 10 years before receiving a diagnosis of dementia. *P* values represent statistical significance of the difference in proportions between groups.

### 3.6 PPV

For calculating PPVs, we included 3943 individuals with dementia and 83,300 controls (without dementia or other chronic neurological diseases) aged >65. Table 4 summarizes the PPVs for the exposures that had evidence of their association with subsequent dementia. Memory difficulties (PPV = 37%) were the strongest predictor followed by hypotension (11.5%) and depression (10.8%).

**TABLE 4:** Positive predictive values among population over 65.

Prediagnostic symptom	Positive predictive value (%)
Depression	10.79
Anxiety	6.28
Use of Antipsychotics	9.59
Insomnia	5.92
Constipation	7.26
Incontinence	8.99
Hypotension	11.47
Imbalance	5.93
Dizziness	5.77
Hearing loss	6.26
Memory	36.92

*Note*: Positive predictive value of each symptom within 10 years is shown in those aged over 65.

## 4 DISCUSSION

### 4.1 Summary of findings

In this study, we used a large primary care dataset to investigate prediagnostic signs and symptoms of dementia in a deprived and highly diverse population. According to the 2021 UK Census, London has the largest ethnic diversity in the UK, with East London having one of the highest rates of deprivation. In a recent study, using the same diverse dataset from London, dementia risk was shown to be higher in individuals from Black and South Asian backgrounds compared to those from White backgrounds,^15^ highlighting the importance of addressing underrepresentation of people from minoritized ethnic backgrounds in dementia research. To our knowledge, this is the first study to investigate prediagnostic presentations of dementia in a diverse population with substantial proportions of people identifying as Black or South Asian.

We explored a range of symptoms recorded in primary care settings up to a decade before diagnosis of dementia. Difficulties with memory were the most common prediagnostic symptom, increasing the odds of receiving a dementia diagnosis in 10 years by approximately 14‐fold. Cognitive decline and, specifically, memory difficulties have been shown to be the most prominent prodromal symptoms in all dementia types.^16^, ^17^ Dementia risk prediction models are mainly focused on cognitive abilities,^18^ and the pathway to receiving a dementia diagnosis in the UK typically starts with a presentation to primary care with a complaint of memory difficulties. Here we show that adults aged >65 from diverse backgrounds have 37% probability of receiving a dementia diagnosis within 10 years after memory difficulty is recorded in primary care.

Besides cognitive changes, a majority of research on prodromal dementia is focused on neuropsychiatric symptoms,^6^, ^19^, ^20^, ^21^ among which we found depression, anxiety, and use of antipsychotics to be associated with increased odds of receiving a dementia diagnosis within 10 years and insomnia to be associated with subsequent dementia within 2 years before receiving the diagnosis. The association between dementia and depression is complex, with some studies pointing toward its being a modifiable risk factor^22^, ^23^, ^24^ and some deeming depression a prodromal symptom.^25^, ^26^, ^27^ In this study, as we got closer to the dementia diagnosis, the association became stronger, providing some evidence for depression occurring for the first time in late life being predominantly associated as a prodromal symptom of incipient dementia.

Among the autonomic symptoms, we found hypotension, incontinence, and constipation to be more common in the years prior to receiving a dementia diagnosis, replicating results of previous longitudinal studies.^6^, ^8^, ^28^, ^29^ Erectile dysfunction was only associated with subsequent dementia 5 to 10 years before the diagnosis. We found individuals experiencing constipation and incontinence had 7.3% and 9% probability of receiving a dementia diagnosis in 10 years, respectively. However, people from ethnic minorities and lower‐income backgrounds may be less likely to seek help for these symptoms, and they are likely to be underreported in these groups,^30^ suggesting that the prevalence and strength of association might be underestimated. In line with existing evidence,^31^ we found hypotension to be associated with increased odds of subsequent dementia, with the odds increasing as we get closer to the dementia diagnosis. Hypotension has been proposed as an early marker of dementia and may reflect shifts in autonomic tone due to focal involvement of the central autonomic control network in early neuropathological change^32^, ^33^ and is one of the diagnostic criteria for prodromal dementia with Lewy bodies.^34^, ^35^ Here we show it to be also prevalent in all‐cause dementia, making it one of the more promising prediagnostic symptoms with a positive predictive value of 11.5%.

Regarding sensory changes prior to dementia diagnosis, we explored the prevalence of musculoskeletal pain including neck and shoulder pain and hearing loss. Musculoskeletal pain associated with lower odds of receiving a dementia diagnosis within 2 to 5 years of diagnosis, and hearing loss was associated with increased odds of receiving a dementia diagnosis within 2 years of diagnosis. The association between hearing loss and dementia has been widely reported.^28^, ^36^, ^37^, ^38^ However, its nature remains unclear. We found hearing loss to be associated only with subsequent dementia within 2 years, in line with recent evidence emphasizing the potential existence of a reverse causal link between hearing loss and dementia and raising the possibility that hearing loss might be an early presentation of dementia as well as a causal risk factor.^39^

We found evidence here that several symptoms were more strongly associated with subsequent dementia diagnosis in Black and South Asian than in White ethnic groups. In particular, both South Asian and Black patients more commonly had recordings of constipation, incontinence, imbalance, and memory difficulties prior to dementia diagnosis. Further work is required to establish the extent to which these reflect altered patterns of help‐seeking behavior that are determined by sociocultural factors or the extent to which they might reflect barriers and delays to recognition by clinicians of a dementia syndrome in those from minoritized ethnic backgrounds. The increased recording of memory difficulties prior to dementia in these ethnic groups provides some evidence in support of the latter interpretation, that is, they may be having more healthcare encounters with problems other than dementia being recorded prior to a dementia diagnosis, even when they present in a typical way with memory loss.

### 4.2 Limitations

This study has several limitations. The cross‐sectional retrospective nature of the data did not allow for calculation of incidence rates and the use of survival modeling. Like other studies using routinely collected primary care data, lack of sufficient data of all variables of interest and rates of missingness posed challenges that could have introduced bias into the analyses. We were unable to look at several candidate factors such as apathy because of low prevalence (possibly reflecting low likelihood of features being recorded in primary care rather than prevalence per se) and had to use proxy measures for psychotic features. However, missingness in ethnicity was low compared to comparable primary care datasets, which allowed this study to be highly representative of the ethnic diversity of the region.

A further limitation is that, due to unreliable recording of dementia subtypes in primary care records, we chose to focus here on all‐cause dementia, which limited our ability to make more biological inferences about the specificity of presenting features for particular clinicopathological syndromes. Moreover, we were not able here to evaluate associations with specific etiologies of the presenting features. For example, there are likely to be differential patterns of association for causes of insomnia such as sleep apnea and REM sleep behavior disorder or for central and peripheral causes of hearing loss. Future work should explore in more detail these associations between specific pathologies associated with both presenting features and dementia syndromes.

## 5 CONCLUSION

In this study, we investigated the signs and symptoms people from diverse backgrounds experience and seek help for in primary care settings in the years prior to receiving a dementia diagnosis. As well as memory difficulties, a range of neuropsychiatric, autonomic, and sensory features were potential early presenting features of dementia in primary care, and the importance of many of these as risk markers varied by ethnicity.

## AUTHOR CONTRIBUTIONS

*Concept and design*: Sedigheh Zabihi, Jonathan P. Bestwick, Christine Carter, Moïse Roche, Sarah Morgan‐Trimmer, Yvonne Birks, Mark Wilberforce, Fiona M. Walter, Claudia Cooper, Charles R. Marshall. *Acquisition, analysis and interpretation of data*: Sedigheh Zabihi, Jonathan P. Bestwick, Mark Jitlal, Phazha L.K. Bothongo, Qiqi Zhang, John Robson, Charles R. Marshall. *Drafting of the manuscript*: Sedigheh Zabihi, Charles R. Marshall. *Critical revision of manuscript for important intellectual content*: Sedigheh Zabihi, Jonathan P. Bestwick, Mark Jitlal, Phazha L.K. Bothongo, Qiqi Zhang, Christine Carter, Moïse Roche, Sarah Morgan‐Trimmer, Yvonne Birks, Mark Wilberforce, Ruth Dobson, Alastair J. Noyce, John Robson, Fiona M. Walter, Claudia Cooper, Charles R Marshall. *Statistical analysis*: Sedigheh Zabihi, Jonathan P. Bestwick, Qiqi Zhang, Charles R. Marshall. *Obtained funding*: Sedigheh Zabihi, Jonathan P. Bestwick, Christine Carter, Moïse Roche, Sarah Morgan‐Trimmer, Yvonne Birks, Mark Wilberforce, Fiona M. Walter, Claudia Cooper, Charles R. Marshall. *Administrative, technical or material support*: Ruth Dobson, Alastair J. Noyce. *Supervision*: Charles R. Marshall.

## ADDITIONAL CONTRIBUTIONS

We are grateful to the general practitioners and patients in East London for the use of data from their electronic health records and the Clinical Effectiveness Group, Queen Mary University of London, who provided a deidentified curated extract of the relevant coded data. The code sets used were supported by the Secure Health Analysis and Research in East London study funded by Barts Charity.

## CONFLICT OF INTEREST STATEMENT

Authors declare no conflicts of interest. Author disclosures are available in the supporting information.

## CONSENT STATEMENT

Due to the anonymized nature of the dataset, consent was not needed.

## Supporting information



Supporting Information

Supporting Information

## Data Availability

Data are not available to share.
