# Supplementary material for: Early presentations of dementia in a diverse population
Source: Alzheimers Dement. 2025 Feb 26;21(2):e14578. doi: 10.1002/alz.14578 (PMC11863067; doi:10.1002/alz.14578)
Supplement: Supplementary file 1 — Supporting Information [file ALZ-21-e14578-s001.docx]

# Supplementary materials

| **Table S1- Selected exposures for inclusion in analysis** | |
| --- | --- |
| Selected Exposures | Depression, Anxiety, Use of antipsychotics, Insomnia, Apathy,  Incontinence, Constipation, Hypotension, Dizziness, Fatigue,  Erectile dysfunction, Balance difficulties, Weight loss, Hearing loss, Tremor,  Headache, Shoulder Pain, Neck pain, Memory difficulties |

| **Table S2- Diagnostic codes for neurological conditions** | |
| --- | --- |
| **Diagnosis** | **EMIS Read Codes** |
| Dementia QOF* code | Eu02%, E00%, Eu01%, E02y1, E012%, Eu00%, E041, Eu041, F110, F111, F112, F116, F118, F21y2, A411%, A410, Eu107, F11x7 |
| Parkinson’s disease | F12%, F1303, F11x9,147F |
| Atypical Parkinson’s disease | F24y0, F24y2, F11y2, F174 |
| Multiple Sclerosis | F20% |
| Motor Neuron disease/ALS | F152% |

| **Table S3- Diagnostic codes for exposures** | |
| --- | --- |
| **Exposure** | **EMIS Read Codes** |
| Depression | E0013, E0021, E112.% , E113.%, E118. , E11y2 , E11z2 , E130. , E135. , E2003 , E291. , E2B.. , E2B1. , Eu204 , Eu251 , Eu32.%, Eu33.% , Eu341 , Eu412  Exclusion: Eu32A , Eu32B , Eu329 |
| Anxiety | 1B1, 1B12, 1B13, 1B1V, 1BK, E200%, Eu41, Eu410, Eu411, Eu412, Eu41z, R2y2 |
| Insomnia | 1B1B%, 1B1Q, 1BX0, E2741, E2742, E274D, Fy00, R005 |
| Apathy | 1BP, 1BP0, 2254, EMISL01, HAYTOLO2, R00z7 |
| Fatigue | 168, 1682, 1683, 1684, 168Z, E205, Eu460, R0071, R0073, R0075 , R007z |
| Constipation | 19C, 19C2, 19CZ, J5200, J5201, J5202, J5204, J520z |
| Incontinence | 16F, 1A23, 1A24, 1A24-1, 1A25, 1A25-1, 1A26, 3940, 8C14, 8C14-1, K586, R083, R083z |
| Erectile dysfunction | 1598, 1ABB, 1ABC, 1D1B, 7C25E, E2273 |
| Hypotension | 14AS, G87, G870, G871, G872, G87z , Gyu90, R1y3, 1B55 |
| Balance difficulties | 1B5, 1B52 , 29L8 , 29LB , 29LD , 29LF , 2994, 2987 |
| Tremor | 1B22 , 297A , 297B , R0103, R20-1 (syn) |
| Vision difficulties | Referral to optometrist: 8H7H, 8H1C |
| Musculoskeletal pain | Neck pain: 16A% (exclude 16A1), N131 , N135z, N138  Shoulder pain: N0941, N0942, N094A, N0951, N210, N2457 |
| Migraines | BARTOCL13, EMISNQCH57, ESCTHE7, F26, F260, F260-1, F261, F261-1, F2610, F2611, F261z, F262, F2620, F2621, F2622, F2623, F2624, F2625, F2626, F2627, F2628, F2629, F262A, F262B, F262C, F262D, F262E, F262z, F26y, F26y0, F26y1, F26y2. F26y3, F26yz, F26z, OX791HP |
| Hearing loss | Deafness code: F59%, 2BL%, SJ15%, P40z%, A5602, 1C13% Audiology referrals: 8HT3 Referral to audiology clinic, 9N0W Seen in audiology clinic, 7P12% Diagnostic audiology |
| Memory symptoms | 1B1A, 1B1Y , 1B1a , 1S21 , 28G, 3A10 , 3A20 , 3A30 , 3A40 , 3A50 , 3A60 , 3A70 , 3A80 , 3A91 , 3AA1 , 8BIk , 8HTY , 9Nk1 , E2A10, E2A11, R00z0 |
| Dizziness | 1B5, 1B53, 1B54, R004, R0040, R0041, R0042, R004z |

| **Table S4- Matched Case-Control Analysis of Pre-diagnostic Signs and Symptoms Adjusted for deprivation (IMD)** | | | | |
| --- | --- | --- | --- | --- |
| **Category** | Time period | | | |
|  | <2 | 2 to<5 years | 5 to 10 years | <10 years |
|  | OR (95% CI) | OR (95% CI) | OR (95% CI) | OR (95% CI) |
| **Pre-diagnostic signs and symptoms** | | | | |
| ***Neuropsychiatric*** | | | | |
| Depression | 5.88 (4.35 to 7.93) *** | 2.51 (1.86 to 3.40) *** | 1.78 (1.41 to 2.24) *** | 2.66 (2.28 to 3.11) *** |
| Anxiety | 3.07 92.39 to 3.94) *** | 1.35 (1.03 to 1.78) * | 1.31 (0.6 to 1.62) * | 1.68 (1.46 to 1.93) *** |
| Use of Antipsychotics | 12.32 (8.72 to 17.41) *** | 3.88 (2.64 to 5.72) *** | 3.14 (2.22 to 4.44) *** | 5.78 (4.73 to 7.04) *** |
| Insomnia | 1.72 (1.34 to 2.22) *** | 1.08 (0.85 to 1.37) | 1.19 (1.00 to 1.41) * | 1.25 (1.11 to 1.42) *** |
| ***Autonomic*** | | | | |
| Fatigue | 0.92 (0.71 to 1.20) | 1.13 (0.90 to 1.41) | 1.26 (1.05 to 1.52) * | 1.13 (0.99 to 1.29) |
| Constipation | 1.70 (1.40 to 2.05) *** | 1.36 (1.15 to 1.62) *** | 1.58 (1.36 to 1.83) *** | 1.54 (1.39 to 1.71) *** |
| Erectile Dysfunction | 0.95 (0.67 to 1.34) | 1.27 (0.99 to 1.63) | 1.25 (1.00 to 1.55) * | 1.19 (1.02 to 1.39) * |
| Incontinence | 3.28 (2.70 to 3.98) *** | 2.73 (2.20 to 3.39) *** | 2.05 (1.65 to 2.55) *** | 2.7 (2.38 to 3.06) *** |
| Hypotension | 3.34 (2.43 to 4.60) *** | 1.94 (1.39 to 2.71) *** | 1.31 (0.89 to 1.92) | 2.12 (1.74 to 2.59) *** |
| ***Sensory*** | | | | |
| Musculoskeletal Pain | 0.88 (0.70 to 0.94) | 0.81 (0.68 to 0.97) * | 0.92 (0.80 to 1.05) | 0.88 (0.79 to 0.97) * |
| Hearing loss | 1.42 (1.18 to 1.70) *** | 1.09 (0.90 to 1.32) | 1.06 (0.88 to 1.27) | 1.17 (1.05 to 1.31) ** |
| Balance difficulties | 1.33 (1.10 to 1.61) ** | 1.42 (1.21 to 1.68) *** | 1.21 (1.03 to 1.44) * | 1.32 (1.18 to 1.46) *** |
| Dizziness | 1.50 (1.25 to 1.80) *** | 1.31 (1.12 to 1.54) ** | 1.16 (1.00 to 1.33) * | 1.28 (1.16 to 1.41) *** |
| ***Cognitive*** | | | | |
| Memory difficulties | 97.02 (79.56 to 118.31) *** | 26.21 (20.73 to 33.14) *** | 14.62 (10.54 to 20.28) *** | 54.56 (46.75 to 63.67) *** |

|  | | | | | | | | | | | |
| --- | --- | --- | --- | --- | --- | --- | --- | --- | --- | --- | --- |
| **Table S5- Results of unmatched analysis including cases and matched controls** | | | | | | | | | | | |
|  | Time period | | | | | | | | | | |
|  | <2 | | 2 to<5 years | | | 5 to 10 years | | | <10 years | | |
| Category | Unadjusted | Adjusted | Unadjusted | Adjusted | Unadjusted | | Adjusted | Unadjusted | | Adjusted |  |
| **Pre-diagnostic signs and symptoms** | | | | | | | | | | | |
| ***Neuropsychiatric*** | | | | | | | | | | | |
| Depression | 6.15 (4.59 to 8.25) *** | 1: 6.37 (4.75 to 8.56) ***  2: 6.11 (4.53 to 8.23) *** | 2.46 (1.83 to 3.29) *** | 1: 2.56 (1.91 to 3.43) ***  2: 2.54 (1.89 to 3.41) *** | 1.765 (1.41 to 2.21) *** | | 1: 1.80 (1.43 to 2.25) ***  2: 1.79 (1.43 to 2.24) *** | 2.66 (2.29 to 3.10) *** | | 1: 2.74 (2.35 to 1.19) ***  2: 2.70 (2.31 to 3.14) *** |  |
| Anxiety | 2.95 (2.31 to 3.76) *** | 1: 2.98 (2.33 to 3.81) ***  2: 3.02 (2.36 to 3.87) *** | 1.33 (1.02 to 1.74) * | 1: 1.34 (1.03 to 1.75) *  2: 1.33 (1.02 to 1.75) * | 1.29 (1.05 to 1.58) * | | 1: 1.30 (1.05 to 1.59) *  2:1.30 (1.05 to 1.60) * | 1.64 (1.43 to 1.89) *** | | 1: 1.66 (1.44 to 1.90) ***  2: 1.66 (1.44 to 1.91) *** |  |
| Use of Antipsychotics | 12.32 (8.86 to 17.13) *** | 1: 12.64 (9.08 to 17.59) ***  2: 12.34 (8.85 to 17.21) *** | 3.86 (2.65 to 5.61) *** | 1: 4.08 (2.80 to 5.95) ***  2: 4.03 (2.76 to 5.89) *** | 3.02 (2.16 to 4.23) *** | | 1: 3.13 (2.24 to 4.37) ***  2: 3.13 (2.24 to 4.39) *** | 5.60 (4.62 to 6.79) *** | | 1: 5.81 (4.79 to 7.05) ***  2: 5.73 (4.72 to 6.96) *** |  |
| Insomnia | 1.67 (1.30 to 2.15) *** | 1: 1.67 (1.30 to 2.15) ***  2: 1.70 (1.32 to 2.19) *** | 1.11 (0.87 to 1.40) | 1: 1.09 (0.86 to 1.38)  2: 1.10 (0.87 to 1.40) | 1.19 (1.01 to 1.41) * | | 1: 1.17 (0.99 to 1.38)  2: 1.20 (1.02 to 1.42) * | 1.25 (1.11 to 1.42) *** | | 1: 1.24 (1.09 to 1.40) **  2: 1.26 (1.12 to 1.43) *** |  |
| ***Autonomic*** | | | | | | | | | | | |
| Fatigue | 0.89 (0.69 to 1.16) | 1: 0.89 (0.68 to 1.16)  2: 0.91 (0.70 to 1.19) | 1.14 (0.92 to 1.43) | 1: 1.15 (0.92 to 1.43)  2: 1.14 (0.91 to 1.42) | 1.23 (1.03 to 1.48) * | | 1: 1.23 (1.02 to 1.48) *  2: 1.24 (1.03 to 1.49) * | 1.12 (0.98 to 1.27) | | 1: 1.12 (0.98 to 1.27)  2: 1.12 (0.99 to 1.28) |  |
| Constipation | 1.69 (1.41 to 2.04) *** | 1: 1.68 (1.39 to 2.02) ***  2: 1.68 (1.39 to 2.02) *** | 1.39 (1.17 to 1.64) *** | 1: 1.37 (1.16 to 1.63) ***  2: 1.37 (1.1 to 1.62) *** | 1.58 (1.37 to 1.83) *** | | 1: 1.57 (1.35 to 1.81) ***  2: 1.56 (1.36 to 1.83) *** | 1.54 (1.40 to 1.71) *** | | 1: 1.53 (1.38 to 1.69) ***  2: 1.53 (1.38 to 1.69) *** |  |
| Erectile Dysfunction | 0.95 (0.67 to 1.34) | 1: 0.96 (0.67 to 1.35)  2: 0.97 (0.68 to 1.37) | 1.21 (0.94 to 1.55) | 1: 1.21 (0.95 to 1.56)  2: 1.24 (0.96 to 1.59) | 1.21 (0.97 to 1.49) | | 1: 1.21 (0.98 to 1.50)  2: 1.20 (0.97 to 1.48) | 1.16 (0.99 to 1.35) | | 1: 1.16 (0.99 to 1.36)  2: 1.16 (0.99 to 1.36) |  |
| Incontinence | 3.15 (2.61 to 3.80) *** | 1: 3.15 (2.61 to 3.80) ***  2: 3.26 (2.70 to 3.94) *** | 2.70 (2.20 to 3.32) *** | 1: 2.71 (2.20 to 3.34) ***  2: 2.83 (2.30 to 3.49) *** | 2.07 (1.67 to 2.56) *** | | 1: 2.07 (1.68 to 2.57) ***  2: 2.10 (1.69 to 2.60) *** | 2.63 (2.34 to 2.97) *** | | 1: 2.64 (2.34 to 2.98) ***  2: 2.72 (2.41 to 3.08) *** |  |
| Hypotension | 3.32 (2.43 to 4.52) *** | 1: 3.28 (2.41 to 4.48) ***  2: 3.31 (2.42 to 4.52) *** | 1.91 (1.38 to 2.66) *** | 1: 1.89 (1.36 to 2.62) ***  2: 1.95 (1.40 to 2.72) *** | 1.32 (0.91 to 1.92) | | 1: 1.30 (0.89 to 1.89)  2: 1.30 (0.89 to 1.90) | 2.11 (1.74 to 2.55) *** | | 1: 2.08 (1.71 to 2.52) ***  2: 2.11 (1.73 to 2.56) *** |  |
| ***Sensory*** | | | | | | | | | | | |
| Imbalance | 1.29 (1.07 to 1.56) ** | 1: 1.28 (1.06 to 1.55) **  2: 1.31 (1.08 to 1.58) ** | 1.44 (1.22 to 1.69) *** | 1: 1.41 (1.20 to 1.66) ***  2: 1.43 (1.21 to 1.68) *** | 1.20 (1.02 to 1.41) * | | 1: 1.19 (1.01 to 1.40) *  2: 1.19 (1.00 to 1.40) * | 1.31 (1.18 to 1.45) *** | | 1: 1.29 (1.16 to 1.43) ***  2: 1.30 (1.17 to 1.45) *** |  |
| Musculoskeletal Pain | 0.87 (0.69 to 1.10) | 1: 0.87 (0.60 to 1.10)  2: 0.88 (0.70 to 1.10) | 0.82 (0.69 to 0.98) * | 1: 0.82 (0.68 to 0.98) *  2: 0.82 (0.69 to 0.98) * | 0.91 (0.79 to 1.04) | | 1: 0.90 (0.79 to 1.03)  2: 0.91 (0.80 to 1.04) | 0.87 (0.79 to 0.97) * | | 1: 0.87 (0.79 to 0.96) **  2: 0.88 (0.79 to 0.97) *_ |  |
| Hearing loss | 1.39 (1.17 to 1.66) *** | 1: 1.36 (1.14 to 1.62) **  2: 1.43 (1.19 to 1.71) *** | 1.12 (0.93 to 1.35) | 1: 1.09 (0.91 to 1.31)  2: 1.12 (0.93 to 1.35) | 1.08 (0.91 to 1.29) | | 1: 1.06 (0.88 to 1.26)  2: 1.06 (0.89 to 1.27) | 1.19 (1.07 to 1.33) ** | | 1: 1.16 (1.04 to 1.30) **  2: 1.19 (1.07 to 1.33) ** |  |
| Dizziness | 1.44 (1.21 to 1.72) *** | 1: 1.43 (1.20 to 1.70) ***  2: 1.46 (1.22 to 1.74) *** | 1.30 (1.11 to 1.52) ** | 1: 1.28 (1.10 to 1.50) **  2: 1.31 (1.12 to 1.53) ** | 1.14 (0.99 to 1.31) | | 1: 1.12 (0.98 to 1.29)  2: 1.14 (0.99 to 1.31) | 1.26 (1.15 to 1.38) *** | | 1: 1.24 (1.13 to 1.37) ***  2: 1.26 (1.15 to 1.39) *** |  |
| ***Cognitive*** | | | | | | | | | | | |
| Memory difficulties | 64.01 (56.01 to 73.16) *** | 1: 64.39 (56.33 to 73.62) ***  2: 68.34 (59.64 to 78.30) *** | 20.05 (16.73 to 24.01) *** | 1: 20.10 (16.77 to 24.08) ***  2: 21.59 (17.97 to 25.94) *** | 12.97 (9.96 to 16.88) *** | | 1: 13.03 (10.01 to 16.97) ***  2: 13.57 (10.38 to 17.74) | 40.73 (36.65 to 45.27) *** | | 1: 40.91 (36.80 to 45.49) ***  2: 43.41 (38.96 to 48.37) *** |  |

The association between experiencing signs and symptoms and receiving a dementia diagnosis within 2, 2 to 5 and 5 to 10 years later is shown in odds ratios (95% confidence intervals); P-value: * p<0.05; ** p<0.01, ***p<0.001

1: adjusted for age and gender

2: adjusted for age, gender and IMD

Total N for erectile dysfunction: 8,043

| **Table S6- Results of unmatched analysis including cases and all controls, stratified by ethnicity** | | | | | | | | | | | | | | | | |
| --- | --- | --- | --- | --- | --- | --- | --- | --- | --- | --- | --- | --- | --- | --- | --- | --- |
|  | **Time period** | | | | | | | | | | | | | | | |
|  | <2 years | | | | 2 to <5 years | | | | 5 to <10 years | | | | <10 years | | | |
| Ethnicity | White | Black | S. Asian | Other | White | Black | S. Asian | Other | White | Black | S. Asian | Other | White | Black | S. Asian | Other |
| **Neuropsychiatric** | | | | | | | | | | | | | | | | |
| Depression | 7.15 (5.21 to 9.79) *** | 5.07 (2.67 to 9.64) *** | 12.39 (7.46 to 20.58) *** | 8.16 (3.53 to 18.89) *** | 2.47 (1.73 to 3.53) *** | 1.58 (0.77 to 3.23) | 3.13 (1.71 to 5.76) *** | 5.35 (2.47 to 11.59) *** | 2.06 (1.55 to 2.72) *** | 2.67 (1.70 to 4.20) *** | 2.25 (1.31 to 3.88) ** | 3.18 (1.53 to 6.60) ** | 2.97 (2.47 to 3.57) *** | 2.71 (1.93 to 3.78) *** | 4.05 (2.94 to 5.57) *** | 4.74 (2.98 to 7.52) *** |
| Anxiety | 3.17 (2.40 to 4.18) *** | 2.59 (1.51 to 4.44) ** | 2.47 (1.43 to 4.26) ** | 2.67 (1.21 to 5.91) * | 1.32 (0.95 to 1.83) | 1.56 (0.84 to 2.88) | 1.80 (1.01 to 3.22) * | 0.70 (0.21 to 2.34) | 1.49 (1.15 to 1.94) ** | 1.55 (0.94 to 2.57) | 1.76 (1.12 to 2.76) * | 2.98 (1.59 to 5.56)** | 1.79 (1.51 to 2.13) *** | 1.82 (1.32 to 2.51) *** | 1.93 (1.42 to 2.63) *** | 2.10 (1.32 to 3.33) ** |
| Use of Antipsychotics | 12.49 (8.97 to 17.40) *** | 14.29 (8.85 to 23.07) *** | 17.83 (11.02 to 28.95) *** | 16.22 (6.80 to 38.72) *** | 4.07 (2.54 to 6.54) *** | 4.14 (2.20 to 7.76) *** | 4.82 (2.57 to 9.06) *** | 5.71 (1.87 to 17.42) ** | 2.43 (1.54 to 3.84) *** | 3.67 (2.15 to 6.26) *** | 4.26 (2.21 to 8.18) *** | 2.77 (1.02 to 7.49) * | 5.49 (4.35 to 6.93) *** | 6.19 (4.52 to 8.48) *** | 7.88 (5.67 to 10.96) *** | 6.08 (3.41 to 10.84) *** |
| Insomnia ± | 0.94 (0.65 to 1.36) | 1.31 (0.80 to 2.13) | 2.34 (1.52 to 3.60) *** | 0.68 (0.15 to 3.12) | 0.55 (0.41 to 0.75) *** | 0.32 (0.18 to 0.56) *** | 0.57 (0.35 to 0.93) * | 0.82 (0.23 to 2.97) | 1.65 (1.30 to 2.10) *** | 1.30 (0.89 to 1.89) | 2.25 (1.63 to 3.12) *** | 2.39 (1.14 to 5.02) | 0.97 (0.82 to 1.16) | 0.83 (0.63 to 1.08) | 1.47 (1.15 to 1.86) ** | 1.49 (0.98 to 2.29) |
| **Autonomic** | | | | | | | | | | | | | | | | |
| Fatigue | 1.12 (0.80 to 1.60) | 0.71 (0.38 to 1.33) | 1.07 (0.64 to 1.80) | 1.14 (0.50 to 2.56) | 1.70 (1.29 to 2.24) *** | 0.76 (0.43 to 1.35) | 0.95 (0.59 to 1.54) | 2.22 (1.24 to 3.97)** | 1.57 (1.23 to 2.00) *** | 1.83 (1.29 to 2.58) | 1.15 (0.78 to 1.67) | 1.28 (0.63 to 2.60) | 1.48 (1.26 to 1.75) *** | 1.18 (0.90 to 1.55) | 1.06 (0.81 to 1.39) | 1.55 (1.03 to 2.33) * |
| Constipation ± | 2.23 (1.71 to 2.90) *** | 1.81 (1.27 to 2.58) ** | 1.58 (1.08 to 2.30) * | 1.77 (0.88 to 3.57) | 1.99 (1.56 to 2.54) *** | 1.67 (1.21 to 2.30) ** | 1.73 (1.26 to 2.36) ** | 0.99 (0.45 to 2.18) | 2.11 (1.70 to 2.61) *** | 1.87 (1.43 to 2.45) *** | 1.56 (1.17 to 2.09) ** | 2.25 (1.45 to 3.49) *** | 2.10 (1.82 to 2.43) *** | 1.79 (1.48 to 2.17) *** | 1.63 (1.33 to 1.99) *** | 1.79 (1.25 to 2.54) ** |
| Erectile Dysfunction | 1.25 (0.73 to 2.14) | 0.72 (0.37 to 1.40) | 1.37 (0.76 to 2.50) | 0.36 (0.5 to 2.70) | 1.04 (0.69 to 1.56) | 0.90 (0.58 to 1.39) | 1.62 (1.10 to 2.39) * | 0.52 (0.18 to 1.45) | 1.12 (0.79 to 1.60) | 1.01 (0.71 to 1.44) | 1.75 (1.21 to 2.53) ** | 0.67 (0.30 to 1.57) | 1.11 (0.87 to 1.43) | 0.92 (0.70 to 1.21) | 1.63 (1.25 to 1.14) *** | 0.57 (0.30 to 1.08) |
| Incontinence ± | 3.21 (2.44 to 4.23) *** | 4.32 (3.11 to 6.01) *** | 4.99 (3.62 to 6.88) *** | 3.30 (1.60 to 6.81) ** | 2.51 (1.90 to 3.31) *** | 3.02 (2.06 to 4.42) *** | 3.39 (2.33 to 4.93) *** | 3.48 (1.83 to 6.62) *** | 2.59 (1.96 to 3.42) *** | 2.23 (1.48 to 3.36) *** | 2.83 (1.87 to 4.26) *** | 2.07 (0.94 to 4.55) | 2.75 (2.33 to 3.25) *** | 3.17 (2.54 to 3.96) *** | 3.75 (2.99 to 4.70) *** | 2.92 (1.91 to 4.48) *** |
| Hypotension ± | 4.74 (3.30 to 6.80) *** | 5.27 (2.91 to 9.54) *** | 2.33 (1.19 to 4.57) * | 1.87 (0.54 to 6.50) | 3.04 (1.98 to 4.67) *** | 4.31 (2.30 to 8.07) *** | 1.79 (0.84 to 3.82) | 4.45 (1.43 to 13.77) * | 2.42 (1.49 to 3.92) *** | 1.01 (0.30 to 3.31) | 2.04 (0.96 to 4.30) | 3.51 91.16 to 10.58) * | 3.41 (2.68 to 4.35) *** | 3.55 (2.39 to 5.30) *** | 2.05 (1.34 to 3.14) ** | 3.05 (1.56 to 5.97) ** |
| **Sensory** | | | | | | | | | | | | | | | | |
| Imbalance ± | 1.34 (1.01 to 1.77) * | 1.75 (1.23 to 2.47) ** | 1.83 (1.28 to 2.60) ** | 1.79 (0.99 to 3.21) | 2.19 (1.77 to 2.71) *** | 1.51 (1.07 to 2.12) * | 1.66 (1.21 to 2.28) ** | 1.90 (1.11 to 3.23) * | 1.89 (1.50 to 2.37) *** | 1.39 (0.99 to 1.93) | 1.75 (1.28 to 2.38) *** | 1.19 (0.63 to 2.24) | 1.82 (1.58 to 2.10) *** | 1.52 (1.24 to 1.87) *** | 1.74 (1.42 to 2.13) *** | 1.60 (1.13 to 2.28) ** |
| Musculoskeletal pain | 0.78 (0.55 to 1.11) | 1.06 (0.68 to 1.66) | 0.69 (0.39 to 1.23) | 0.70 (0.29 to 1.64) | 0.82 (0.63 to 1.08) | 0.93 (0.66 to 1.31) | 1.05 (0.74 to 1.49) | 1.02 (0.56 to 1.84) | 1.02 (0.83 to 1.25) | 0.83 (0.62 to 1.11) | 0.92 (0.68 to 1.23) | 0.84 (0.49 to 1.42) | 0.91 (0.78 to 1.06) | 0.90 (0.73 to 1.11) | 0.92 (0.74 to 1.15) | 0.86 ( 0.59 to 1.26) |
| Hearing loss | 1.66 (1.31 to 2.09) *** | 2.73 (1.93 to 3.86) *** | 1.47 (0.98 to 2.21) | 1.05 (0.47 to 2.34) | 1.48 (1.18 to 1.86) ** | 1.22 (0.76 to 1.94) | 0.81 (0.50 to 1.31) | 1.42 (0.75 to 2.68) | 1.32 (1.03 to 1.69) * | 1.51 (0.97 to 2.36) | 1.80 (1.27 to 2.53) ** | 2.59 (1.45 to 4.61) ** | 1.49 (1.29 to 1.72) *** | 1.81 (1.42 to 2.31) *** | 1.35 (1.06 to 1.72) * | 1.64 (1.11 to 2.43) * |
| Dizziness | 1.36 (1.05 to 1.77) * | 1.79 (1.28 to 2.50) ** | 1.93 91.37 to 2.71) *** | 2.02 (1.17 to 3.49) * | 1.42 (1.13 to 1.79) ** | 1.59 (1.16 to 2.17) ** | 1.77 (1.32 to 2.37) *** | 2.16 (1.33 to 3.49) ** | 1.69 (1.40 to 2.03) *** | 1.19 (0.88 to 1.60) | 1.72 (1.32 to 2.24) *** | 0.96 (0.54 to 1.69) | 1.52 (1.33 to 1.74) *** | 1.46 (1.20 to 1.77) *** | 1.78 (1.48 to 2.15) *** | 1.59 (1.15 to 2.19) ** |
| **Cognitive** | | | | | | | | | | | | | | | | |
| Memory difficulties ± | 90.53 (78.61 to 104.24) *** | 85.20 (69.46 to 104.50) *** | 76.54 (61.60 to 95.10) *** | 73.39 (51.12 to 105.37) *** | 27.73 (22.74 to 33.81) *** | 17.59 (13.07 to 23.67) *** | 16.37 (12.01 to 22.31) *** | 20.64 (12.99 to 32.77) *** | 16.79 (12.54 to 22.49) *** | 14.95 (9.21 to 24.26) *** | 11.07 (6.33 to 19.38) *** | 18.28 (9.14 to 36.55) *** | 53.06 (47.00 to 59.90) *** | 46.69 (39.17 to 55.64) *** | 41.81 (34.31 to 50.94) *** | 39.94 (29.47 to 54.13) *** |

N: white= 442,778; Black= 135,238; S. Asian= 217,093; Other= 114,680; All estimates were adjusted for age and gender, shown as OR (95% CI); *p<0.05, **p<0.01, ***p<0.001; ± evidence found for an interaction with ethnicity
